# Between nostalgia and exclusion: structural constraints and dietary resilience among undocumented Mexican migrants in Los Angeles, California

**DOI:** 10.3389/fnut.2026.1743173

**Published:** 2026-03-04

**Authors:** Pascual García-Macías, Nubia Alejandrina García Bárcenas

**Affiliations:** 1 Universidad Técnica Particular de Loja, Loja, Ecuador; 2 Universidad Autónoma de Zacatecas, Zacatecas, Mexico

**Keywords:** anticipatory governance of fear, dietary transitions, food insecurity, Los Angeles, migration and nutrition, undocumented migrants

## Abstract

Undocumented Mexican migrants in the United States navigate everyday life under conditions shaped not only by economic hardship and legal exclusion, but also by the persistent anticipation of surveillance and immigration enforcement. While existing research often frames dietary change in migration as a result of cultural loss or individual choice, less attention has been paid to how legal precarity and anticipatory fear actively govern migrants’ food practices, health, and mobility. This study examines how undocumented status becomes embodied through everyday food decisions, contributing to broader processes of health vulnerability and structural inequality. This article draws on ethnographic fieldwork conducted among undocumented Mexican migrants in Los Angeles, California. Data were collected through in-depth semi-structured interviews, participant observation recorded in fieldnotes, and photographic diaries documenting everyday food environments and practices. This qualitative approach enabled an examination of how migrants experience, interpret, and navigate food, health, and fear within their daily routines, capturing both material constraints and subjective meanings. The findings reveal that migrants’ dietary practices are shaped not only by limited income and restricted access to nutritious and culturally meaningful foods, but also by what we conceptualize as the anticipatory governance of fear. Even in the absence of direct encounters with immigration authorities, migrants modify their routines to minimize visibility, avoiding certain public spaces, shops, and travel routes. These adaptations often reduce access to healthy foods, contributing to weight gain, fatigue, and chronic health conditions. At the same time, migrants actively preserve traditional foodways, sustain family care through cooking, and create spaces of resilience grounded in memory, cultural continuity, and community ties. These findings demonstrate how undocumented status operates not only as a legal category but as an embodied condition that shapes health outcomes through everyday practices of adaptation, restraint, and survival. By highlighting the anticipatory and lived dimensions of food insecurity, this study challenges individualistic and culturally reductionist explanations of dietary change. Instead, it situates migrant health within broader structures of legal precarity, labor exploitation, and surveillance. This human-centered and ethnographically grounded analysis contributes to critical scholarship on migration, nutrition, and health by revealing how governance, fear, and inequality become inscribed in bodies, diets, and everyday life.

## 1 Introduction

María, a 42-year-old Mexican immigrant who sells tamales on the streets of Los Angeles, vividly recalls an incident that encapsulates the tensions of migration. While selling her homemade tamales, “the flavor of her homeland,” an angry passerby shouted, “Go back to your country!” María confesses: “At first I didn’t understand everything he said, but his tone was enough to make me feel bad,” since she was merely trying to earn a dignified living (María, 42, street vendor). This scene, drawn from fieldwork, illustrates the central axis of this study: migrants live between nostalgia and exclusion. On the one hand, they long for and miss the flavors and culinary culture of home; on the other, they face precariousness, labour exploitation, and rejection within the host society.

Undocumented Mexican migrants in Los Angeles, California, experience profound transformations in their dietary practices alongside health challenges shaped by adverse structural conditions. These dynamics suggest that migrants’ dietary transformations should be examined at the intersection of structural adversity, precarious legality, and food-based cultural resilience. This approach builds upon a growing body of qualitative and ethnographic scholarship that has documented how food practices operate as sites of belonging, memory, and everyday resistance among Mexican migrants in the United States. Previous studies have shown that cooking, selling, and sharing traditional foods allow migrants to sustain transnational identities while navigating exclusionary urban environments (1, 2). Rather than passive markers of nostalgia, food practices often become active strategies through which migrants negotiate dignity, cultural continuity, and economic survival under conditions of legal and social marginalization (3, 4). Situating this study within that literature allows us to foreground migrants’ own perspectives on food as lived experience, while extending existing debates by linking dietary practices to structural vulnerability and health outcomes. This article argues that dietary vulnerability among undocumented Mexican migrants in Los Angeles is shaped not only by structural exclusion and precarious labour conditions, but also by anticipatory fear and early processes of securitization that were already present before the intensification of immigration enforcement under the Trump 2.0 administration. Unlike existing ethnographic studies that examine food practices under already consolidated enforcement regimes, this study captures a pre-second presidential term of Donald Trump moment in which fear of deportation, public visibility, and future repression were already internalized and actively shaping everyday food choices, mobility, and health-related practices. By foregrounding this temporal dimension, the article advances current debates on food insecurity and migration by showing how dietary practices are conditioned by anticipatory governance and lived uncertainty.

To capture these dynamics, we introduce anticipatory governance of fear: the ways the possibility of immigration enforcement shapes everyday routines through self-regulation. We develop the concept in the theoretical framework and use it to interpret how food access, mobility, and health are reorganized under legal precarity (see Table 1).

**Table 1:** Qualitative data collection techniques employed in the study.

Technique	Purpose	Operational details
*Semi-structured interviews* (*n* = 30)	Explore migration trajectories, dietary changes, barriers, and strategies	Expert-validated guide; average duration 40 min; audio-recorded and transcribed
*Participant observation* (~80 h)	Document daily practices of food purchase, preparation, and consumption	Conducted in homes, markets, community kitchens, and workplaces
*Photographic diaries* (*n* = 15)	Capture participants’ visual perceptions of food and health	Taken using mobile phones
*24-h dietary recall* (subsample, *n* = 20)	Describe current intake patterns	Contextualized qualitative narratives

This table summarizes the qualitative data collection techniques employed in the study, including semi-structured interviews, participant observation, photographic diaries, and 24-h dietary recall. Source: Author’s fieldwork and research design, Los Angeles, 2024. All participants gave informed consent; names and identifiable information were replaced by pseudonyms to protect confidentiality.

## 2 Theoretical framework

### 2.1 Structural vulnerability, deportability, and constrained food agency

This study approaches food insecurity among undocumented Mexican migrants not as an outcome of individual choice or cultural adaptation, but as the result of structurally constrained life conditions. Drawing on structural vulnerability (5) and *deportability* (6), it highlights how legal exclusion, labor precarity, and limited access to public institutions systematically shape migrants’ exposure to harm and constrain their ability to maintain healthy diets.

Undocumented status, in this framework, is not simply a legal designation it reorganizes everyday life, shaping how food is accessed, prepared, and consumed. Migrants must navigate low income, time poverty, fear of institutional contact, and exclusion from welfare systems, all of which reduce their actual food agency. Following the capabilities approach (7, 8), food agency here is understood not as formal choice, but as the real opportunity to achieve valued states of health, dignity, and cultural continuity under unequal conditions.

As Carney (9, 10) argues, undocumented migrants are not outside the state; they are conditionally incorporated into morally regulated systems like food aid and fragmented healthcare. This makes food practices a key site to observe how governance, exclusion, and care intersect in everyday life.

### 2.2 Dietary change, nutritional transition, and food environments

Migrant diets often transform drastically after migration. In line with nutritional transition theory (11–13), this shift involves moving from traditional, fresh, plant-based diets toward ultra-processed, calorie-dense foods. These transitions are shaped not by cultural loss alone, but by structural inequalities tied to trade policies, urban segregation, and the global food industry (14, 15).

Concepts such as food apartheid (26) emphasize how low-income and racialized communities face constrained food environments, dominated by fast food and lacking fresh produce. In these contexts, dietary change becomes not a matter of preference, but adaptation to limited and unequal options.

Migrants’ food practices, however, are not passive. As Carney and others (9, 10, 16) suggest, food remains a space of negotiation, care, and moral responsibility. Even amid scarcity, the invocation of traditional foodways signals a struggle for cultural continuity and dignity (2, 17).

### 2.3 Food agency, aspirations, and embodied consequences

To analyze migrants’ food agency under conditions of exclusion, this study draws selectively on the capabilities–aspirations framework (7, 8, 18), not as a predictive model but as a heuristic for understanding how aspirations for health, family care, and cultural preservation persist despite severe structural constraints. For many migrants, however, these aspirations collide with limited capabilities, resulting in compromised dietary practices and adverse health outcomes.

Economic precarity, limited access to fresh and culturally appropriate foods, and exposure to obesogenic environments often lead to increased consumption of inexpensive, energy-dense products, reinforcing patterns of obesity, diabetes, and hypertension documented in epidemiological research (19, 20). These dietary shifts are accompanied by psychosocial consequences, including guilt, stigma, and acculturative stress (21), highlighting that food practices are embedded in broader processes of social valuation and exclusion.

Crucially, this framework understands the body not merely as a biological outcome of dietary change, but as a site where structural exclusion is embodied over time. Building on Carney’s emphasis on food, care, and moral regulation, health outcomes such as weight gain, chronic fatigue, and metabolic illness are read as corporeal expressions of governance operating through everyday food practices.

### 2.4 Anticipatory governance of fear, food practices, and embodied exclusion

This study introduces anticipatory governance of fear as its central analytical lens. Developed in critical dialogue with deportability (6) and structural vulnerability (5), this concept specifies the mechanism through which legal exclusion becomes operative in everyday life. While deportability captures the structural condition of being permanently removable and structural vulnerability highlights unequal exposure to harm, anticipatory governance of fear identifies how these conditions are enacted *before* direct state intervention. Rather than relying on constant surveillance or overt coercion, governance operates through the internalization of threat, leading migrants to regulate their own conduct in advance.

Applied to food practices, anticipatory governance of fear illuminates how migrants adjust where they shop, whether they access food assistance, how long they remain in public spaces, and which routes they take when procuring food. Food insecurity thus emerges not only from economic scarcity or spatial inequality, but from anticipatory self-regulation shaped by fear of exposure and enforcement. This perspective provides a conceptual bridge between structural conditions and lived experience, allowing everyday dietary practices to be read as responses to governance that operates before enforcement.

Importantly, this framework foregrounds the embodied consequences of anticipatory governance. Compromised dietary practices are accompanied by psychosocial effects such as guilt, stigma, and acculturative stress, as well as by physiological outcomes including weight gain, chronic fatigue, hypertension, and metabolic illness. Building on Carney’s work (9, 10, 22), the body is understood here as an archive of governance, where legal precarity, moral regulation, and nutritional constraint sediment over time. Dietary change and its health sequelae are therefore not merely biomedical phenomena, but corporeal expressions of structural exclusion.

These dynamics unfold within food environments marked by profound inequality. Migrants’ reflections on their neighborhoods echo the food apartheid framework (15, 26), which highlights racialized and class-based segregation in access to nutritious foods. In such contexts, even when migrants aspire to eat healthily, their options are constrained by cost, availability, and time poverty. These constraints are intensified by labor precarity and undocumented status, which restrict access to social protection systems and deter engagement with health and food assistance institutions (6, 9).

**Figure 1:**
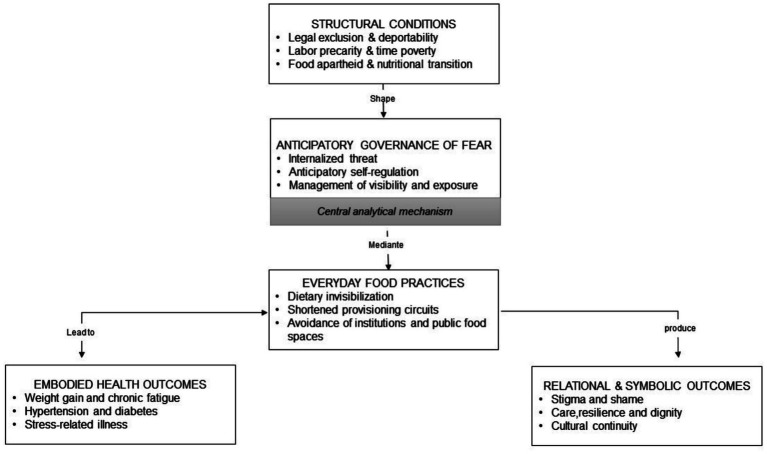
Anticipatory governance of fear and everyday food practices among undocumented Mexican migrants. This figure illustrates how structural conditions, including legal exclusion, labor precarity, food apartheid, and nutritional transition, shape migrants’ everyday food practices through anticipatory governance of fear. Rather than operating through direct enforcement, governance functions via anticipatory self-regulation, influencing dietary invisibilization, shortened food provisioning circuits, and avoidance of institutions. These practices produce embodied health outcomes and psychosocial effects over time, while also giving rise to forms of care, resilience, and cultural continuity. The model highlights how food practices constitute a key site where structural exclusion, governance, and agency intersect in everyday life. Source: Author’s elaboration, integrating capabilities theory (7, 8), deportability (6), and structural vulnerability (5), and extending these frameworks through the concept of *anticipatory governance of fear* to explain how legal exclusion becomes embodied in everyday food practices and health outcomes under conditions of nutritional transition (12).

The outcomes of these negotiations operate on two interrelated planes. On the one hand, physical and mental health are shaped by dietary change, chronic disease, and acculturative stress (20, 21). On the other hand, symbolic and relational well-being is affected through experiences of stigma, loss, resilience, and cultural reproduction. Community networks, informal markets, urban gardens, and food-based solidarities function as fragile infrastructures that can both buffer and reproduce vulnerability (23, 27). Together, this theoretical framework enables an integrated reading of how food practices among undocumented Mexican migrants constitute a field where structural exclusion is reproduced, negotiated, and, at times, resisted in everyday life. Figure 1 synthesizes the analytical framework of the study, showing how structural exclusion operates through anticipatory governance of fear to shape everyday food practices and embodied health outcomes among undocumented Mexican migrants in Los Angeles.

In sum, this theoretical framework brings together critical concepts of structural vulnerability, deportability, and the original lens of anticipatory governance of fear to explain how undocumented migrants’ food practices are shaped by both material constraints and anticipatory self-regulation. By integrating the capabilities–aspirations perspective, the study emphasizes how health, care, and cultural continuity are pursued under constrained conditions. This conceptual model foregrounds the temporal, embodied, and micro-political dimensions of food insecurity and positions food not merely as a nutritional need, but as a key site of governance, agency, and resilience. It sets the stage for analyzing how everyday dietary choices become entangled with the lived experience of legal exclusion in Los Angeles.

## 3 Methodology

### 3.1 Approach and design

This study adopts a qualitative, phenomenological interpretive approach (28), complemented by insights from critical ethnography (29). We aim to understand migrants’ lived experiences of food and health as they are felt, narrated, and practiced in everyday life, while also situating these experiences within the broader power relations that shape them. This interdisciplinary design makes it possible to bring together an emic perspective capturing participants’ own meanings, interpretations, and narratives with an etic reading informed by critical social theory, attentive to structural conditions, inequality, and constraint. Methodologically, this design is suited to examining how governance and legal precarity become lived through mundane routines, such as shopping, cooking, avoiding institutions, and managing visibility, making it particularly appropriate for analyzing anticipatory governance of fear as an everyday mechanism rather than an abstract construct. The ethnographic fieldwork was conducted in Los Angeles, California, between January and August 2024. This timeframe represents a critical socio-political hiatus: the eve of the 2025 political shift. Although these data precede the subsequent intensification of ICE enforcement and the mass social mobilizations observed in 2025, the study captures a state of ‘anticipatory governance of fear’. At this threshold, migrants already operated under a self-imposed paralysis, demonstrating that the fear of visibility and the restriction of mobility were already deeply rooted as defensive mechanisms before the formal change in administration.

Throughout the article, we use the term migrant as an inclusive analytical category to refer to individuals who have moved away from their place of usual residence, without presuming permanence, legal status, or intentions of long-term settlement. The term immigrant is employed only when referring to formal legal classifications or when participants explicitly describe themselves using that label.

### 3.2 Participants and sampling

Thirty undocumented Mexican migrants (17 women and 13 men) residing in the Los Angeles metropolitan area participated. Inclusion criteria were being over 18 years of age, residing in the United States for at least 12 months, and employment primarily in low-wage sectors (cleaning, food service, urban agriculture, construction, or street vending). Sampling began through Latino community organizations and expanded via participant referrals, following theoretical and snowball logic (30). Sample size was determined by theoretical saturation (31), ceasing recruitment once new data no longer contributed to additional analytic categories. Interviews were in-depth and semi-structured, lasting approximately 45–120 min, and were audio-recorded when participants agreed; otherwise, detailed contemporaneous notes were taken. All interviews were transcribed verbatim in Spanish before analysis.

### 3.3 Fieldwork procedure

Fieldwork was conducted from January to August 2024 across several Los Angeles neighborhoods with large Mexican populations, primarily East Los Angeles, Boyle Heights, Montebello, and South Los Angeles. We focused on East Los Angeles and Boyle Heights because these neighborhoods combine dense Mexican-origin settlement with highly visible street-level enforcement anxieties, and because everyday food provisioning involves navigating a mix of ethnic markets, discount retailers, and institutional food assistance sites within short geographic distances. Los Angeles constitutes a historically and structurally significant site for this study, as it is one of the largest destinations for Mexican-origin migration in the United States, a central node in agro-food labor circuits, and a city marked by deep urban inequality and uneven incorporation of undocumented populations. Its long history of migration, combined with intensified immigration enforcement and racialized food environments, makes it a key setting for examining the intersections of diet, fear, and structural vulnerability. The field team included the principal author and a bilingual Mexican migrant assistant (Spanish–English). Access to participants was facilitated through a local church, a trusted acquaintance, and a hometown association that served as community bridges. Informed consent was obtained from all participants after explaining the study’s objectives, assuring confidentiality, and emphasizing their voluntary participation and right to withdraw at any time. Pseudonyms were used, and any identifying data potentially revealing migration status were excluded. Interviews were conducted in Spanish (shared mother tongue) in locations chosen by participants for comfort and privacy, mainly homes, parish halls, parks, or sometimes on the street. A member-checking session was offered to those wishing to review preliminary findings, though fear of deportation limited participation in most cases.

### 3.4 Data analysis and rigor

Data were analyzed using reflexive thematic analysis (32) supported by ATLAS.ti 9. Coding proceeded iteratively from initial descriptive coding to more focused analytic themes, guided by iterative comparison across interviews, fieldnotes, and photographic diaries (31, 32), until analytic themes were sufficiently developed and refined. Transferability was ensured through thick description of context, participants, and observed situations. Dependability was maintained through detailed audit trails of transcripts, analytical notes, coding iterations, and methodological decisions, externally reviewed for consistency. Confirmability was achieved through continuous reflexivity and correlation of interpretations with textual evidence to ensure data-driven conclusions.

### 3.5 Ethical considerations

The study followed internationally recognized ethical principles for social research with vulnerable populations and complied with the ethical standards and data-protection regulations of the authors’ home institutions, including the Mexican Health Research Law (Article 13) and the Federal Law on the Protection of Personal Data in Possession of Private Parties (Article 8, Official Gazette, July 5, 2010). Participants gave express consent verbally. Confidentiality was maintained by using pseudonyms and non-identifying records. Given the potential legal risks of sharing migration-related experiences, no specific identification data were collected. Participants also received contact information for free legal and mental health support services. Each participant received modest compensation for their time. The research was conducted with deep respect for participants, who were regarded not merely as study subjects but as collaborators whose experiences formed the foundation of this work.

## 4 Results

### 4.1 Economic precarity and inadequate diets

In this section, participants’ testimonies are not approached as illustrative accounts of predefined concepts, but as analytically productive narratives that allow us to interrogate, refine, and situate existing theoretical debates on foodways, migration, and inequality. Drawing on feminist food studies and critical migration scholarship, we treat everyday food practices as sites where agency, moral value, vulnerability, and belonging are actively negotiated under conditions of structural constraint (1, 4, 24). This approach enables us to move beyond descriptive readings and to foreground how migrants’ lived experiences contribute to broader discussions on food, work, and uneven incorporation.

Participants’ narratives revealed that economic precarity and marginalization permeate every aspect of life, including their daily diet. Most reported difficulties maintaining a nutritious diet, directly linked this to low income and job instability. Twenty-five out of thirty reported periods when food “wasn’t enough” or they had to choose lower-quality food to fill stomachs. “Sometimes there is not enough money for the week’s food; we buy what’s cheapest: beans, rice, bread […] meat is often a luxury” (Pedro, 38, day labourer). Common coping strategies included skipping meals, reducing portion sizes, or consuming high-calorie but low-nutrient foods (chips, sweet bread, instant noodles). These practices mitigate hunger temporarily but cause nutritional deficiencies and long-term health issues.

Migrant households also face tensions arising from cultural change and acculturation. One mother shared, *“*My kids want pizza instead of stew… it breaks my heart, but sometimes I give in so they’ll eat,” pointing to generational dynamics that complicate the preservation of traditional foodways. These experiences illustrate acculturative stress as a psychological tension between cultural continuity and adaptation. This dynamic connects with literature on identity negotiation in migration, indicating that food practices are sites of cultural negotiation that reflect broader processes of belonging and adaptation. A significant finding is the psychological tension created by food insecurity. Worrying about having enough to eat compounds labour stress and immigration insecurity. “It’s just constant anxiety[…] thinking how to stretch the paycheck for bills, rent, and sometimes for food,” said Sofía (29, cleaning worker). Mothers often sacrificed their own meals for their children. “I’d just have coffee and bread and save the best for my kids” (Lucía, 42), illustrating the gendered dimension of coping strategies. Food insecurity thus emerges as both a physical condition and an emotional vulnerability affecting dignity and peace of mind.

Dietary changes reflected increased consumption of cheap, highly processed foods. “When living alone, $5 would fill me up at Taco Bell or McDonald’s. I knew it wasn’t healthy, but it was easy and cheap,” said Luis (24, waiter). Many lived on hypercaloric but micronutrient-poor diets, high in refined carbs, sugars, and fats but low in fruits, vegetables, and lean proteins.

**Figure 2:**
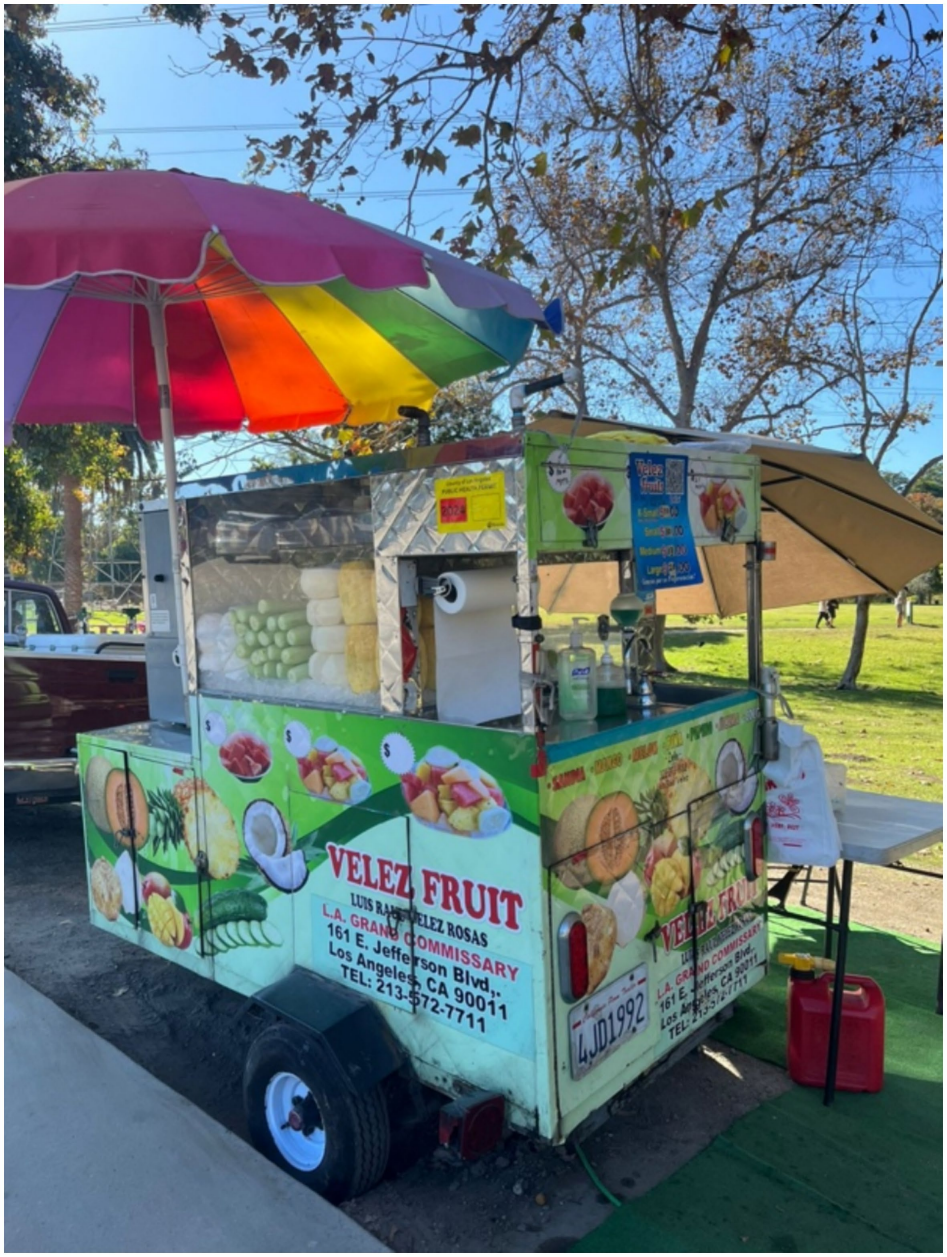
Street fruit vending as a livelihood and cultural resilience strategy. Street vending of fruit among undocumented Mexican migrants in Los Angeles represents both an economic survival mechanism and an act of cultural continuity. The image symbolizes the intersection of precarity, identity, and food agency. Source: Author’s fieldwork, 2024. No identifiable individuals appear; consent and anonymity preserved.

As illustrated in Figure 2, informal street vending of fruit and snacks exemplifies both economic necessity and cultural continuity among undocumented Mexican migrants in Los Angeles.

This “diet of poverty amid affluence” mirrors studies showing that inexpensive foods are calorie-dense yet nutrient-poor. In sum, economic precarity translates into scarce and low-quality diets dominated by cheap calories, forming a vicious cycle where poor diet leads to health problems that remain untreated due to lack of resources.

### 4.2 Anticipatory governance of fear in food access and practices

Beyond material deprivation, participants’ narratives reveal that fear operates as a governing force shaping everyday food-related practices. Across interviews and field observations, migrants described how the anticipation of immigration enforcement structured decisions about where, when, and how to access food. Importantly, these behaviors were not triggered by direct encounters with police or immigration authorities, but by the possibility of such encounters.

Several participants explained that they avoided food banks, churches, or community distribution sites despite experiencing food scarcity. As one participant noted, “I did not go anymore, not because they were there, but because you never know if they might ask for papers.” Others described altering their shopping routines, making faster purchases, avoiding crowded supermarkets, or choosing stores perceived as more “hidden-anonymous,” even when prices were higher, or food quality was lower.

“I prefer to buy less food at the store than to risk going to places where they ask questions.” This anticipatory fear also shaped the research process itself. Fieldwork access to agricultural fields and public spaces was significantly constrained, as many potential participants declined participation due to concerns about visibility and exposure. Despite the absence of overt enforcement actions at the time of research, fear of deportation permeated everyday life, limiting both mobility and willingness to engage with institutions, including academic research.

Fieldnote, Boyle Heights: During some mornings of observation near a Santa Isabel church-based food distribution site, and during some community activities, the line shortened abruptly when a police vehicle passed nearby. Although no officers approached the entrance, several people quietly left the area, dispersing into side streets without collecting food. These accounts illustrate how anticipatory governance of fear operates as a micro-biopolitical mechanism that reorganizes food access through self-regulation. Food insecurity, in this sense, is not only experienced as hunger or dietary compromise but as a condition produced through restraint, avoidance, and constant calculation of risk. The cumulative effect of these practices contributes to stress, disrupted eating patterns, and long-term health vulnerability, reinforcing the embodied consequences of legal exclusion even in moments of relative material stability. These practices demonstrate that food insecurity is produced not only through material scarcity but through anticipatory governance that restricts access before enforcement occurs.

### 4.3 Dietary invisibilization and the management of exposure

A second key finding concerns what we term dietary invisibilization practices: a set of strategies through which undocumented migrants deliberately modify food-related behaviors in order to reduce social and institutional visibility. These practices include accelerated shopping routines, avoidance of public eating spaces, delayed meal times, and a preference for food environments perceived as discreet or familiar, even when this entails nutritional compromise. Rather than being marginal or incidental, dietary invisibilization emerges as a patterned response to anticipatory fear, shaping how, where, and when food is accessed and consumed.

Across interviews, participants emphasized that “things are eaten differently here,” describing a post-migration shift toward the U.S. food supply driven less by preference than by necessity. Most reported increased consumption of industrial and ultra-processed foods alongside a decline in fresh, home-cooked meals. Approximately 60% reported noticeable weight gain after migration, often between 10 and 15 kilograms within the first two years, accompanied by diagnoses of hypertension or prediabetes. As Rosa (35) reflected, “They diagnosed me with prediabetes here […] I think it was all the burgers, pizza, and sodas I got used to.” Yet in the same breath, she explained how fear shaped her everyday food routines: “I go to the supermarket very fast. I grab what I need and leave. I don’t like to stay there.” Her account illustrates how dietary change is entangled with strategies of exposure management rather than with simple acculturation.

Ethnographic observation further confirms these patterns. Fieldnote, East Los Angeles: several participants described eating meals alone in their cars, in backyards, or waiting until late evening to eat at home. Public eating spaces, such as parks, food courts, or workplace cafeterias, were frequently described as “unsafe” or “too exposed,” despite the absence of formal surveillance. These practices amount to what can be described as a diet of exclusion, in which food choices and eating routines are reorganized around invisibility and self-protection rather than nutrition, taste, or sociality.

Maintaining traditional Mexican diets under these conditions proved especially difficult. Long working hours, time poverty, and limited access to culturally specific ingredients pushed meals away from home-cooked beans, corn tortillas, and fresh preparations toward bread, white rice, processed meats, and sugary drinks. Many participants associated these changes with feeling heavier, more fatigued, and less healthy. At the same time, moments of adaptive resilience and learning also emerged. Lucía (42) described removing sodas and junk food from her household following her husband’s hypertension diagnosis, replacing them with natural drinks. Rosa, similarly, learned to incorporate vegetables into every meal as her awareness of diet-related health risks grew. These experiences suggest that acculturation is not a linear trajectory of nutritional decline, but a contingent process in which health awareness and dietary innovation can emerge when minimal stability and information are available.

Following Abarca’s (1) notion of voices in the kitchen, domestic food practices, particularly women’s cooking, emerge as sites where cultural authority, care, and dignity are actively negotiated. Recurrent concerns about “chemicals” and “hormones” in U.S. food reflect deep mistrust of industrial agriculture and its effects on bodies. As María (42) explained, “They put too many hormones in the meat; my daughters developed too fast.” In response, some participants cultivated small herbs such as epazote, cilantro, and chilies in pots, seeking access to what they described as “clean” and “authentic” flavors. These practices reflect both resistance to and adaptation within the industrial food environment.

Read through this lens, cooking does not function merely as a nostalgic attachment to the past. Instead, it emerges as a situated practice of agency through which women reassert moral worth, cultural competence, and continuity in contexts marked by rupture and exclusion. By deciding what enters the household, how it is prepared, and when it is consumed, women actively counter experiences of social devaluation and invisibility. In this sense, culinary practice operates as a form of everyday resistance, echoing Abarca’s argument that the kitchen is not a space of silence but a space of voice.

### 4.4 Shortened food provisioning circuits and constrained food agency

A third key finding concerns the emergence of shortened food provisioning circuits, a pattern through which undocumented Mexican migrants restrict food procurement to a limited set of trusted routes, vendors, and commercial spaces perceived as safe. Rather than navigating the full range of food options available in the urban environment, participants described relying on a narrow and repetitive geography of provisioning shaped by familiarity, social recognition, and the minimization of exposure to perceived risk. These circuits are not merely matters of convenience; they represent adaptive responses to anticipatory fear and legal vulnerability that reorganize food agency in everyday life.

Interview data reveal how trust and predictability become central criteria in food access decisions. As Héctor (44, gardener) explained, “I always go to the same store. I know the people there. I don’t change.” Similarly, Sofía (29, cleaning worker) noted, “I buy what’s close, what I know. I don’t explore other places.” These accounts indicate that food choices are shaped less by price, quality, or nutritional value than by the perceived safety of familiar environments. Even when alternative food outlets were available, participants often avoided them due to uncertainty and fear of visibility. As Luis (24, waiter) reflected, “Sometimes it’s more expensive, but I feel calmer,” highlighting how emotional security is prioritized over economic efficiency or dietary diversity.

Ethnographic observation and photographic diaries further corroborate this pattern. Visual records consistently showed repeated purchases from the same small convenience stores or neighborhood shops, typically offering a limited range of fresh produce and a high concentration of ultra-processed foods. This occurred despite the physical proximity of larger supermarkets with broader and more affordable food options within walking distance. These micro-geographies of provisioning reveal how anticipatory fear contracts migrants’ spatial and nutritional horizons, producing routinized consumption practices anchored in safety rather than choice.

Analytically, shortened food provisioning circuits illustrate how anticipatory governance of fear reshapes food agency by narrowing the field of possible action. While these circuits reduce perceived exposure to surveillance or unwanted attention, they simultaneously constrain dietary diversity and reinforce reliance on inexpensive, energy-dense, and nutritionally poor foods. Food agency, in this sense, does not disappear but becomes strategically bounded, operating within circuits that privilege calm and invisibility over nutritional adequacy. These findings underscore that dietary outcomes among undocumented migrants are not simply the result of limited resources or individual preferences, but of governance processes that act in advance, shaping everyday practices through fear, anticipation, and self-regulation.

Taken together, these findings show that food insecurity among undocumented Mexican migrants in Los Angeles is not solely the outcome of limited income or constrained food environments. Rather, it is produced through a set of everyday practices shaped by anticipatory fear, including dietary invisibilization and shortened food provisioning circuits, which reorganize food access, mobility, and health over time. These patterns invite a broader analytical discussion of how fear operates as a mode of governance that mediates migrants’ relationships with the state and becomes materially and corporeally embedded in everyday life. In the following section, we situate these findings in dialogue with existing scholarship, particularly Carney’s work on food, governance, and embodiment, to examine how anticipatory governance of fear is lived, negotiated, and inscribed on migrant bodies.

## 5 Discussion

This study contributes to scholarship on food, migration, and health by shifting the analytical focus from institutional food environments alone to the everyday mechanisms through which undocumented Mexican migrants navigate food access under conditions of legal precarity. While previous research has shown how immigrant foodscapes are shaped by urban transformation, exclusion, and gentrification (3), and how migrant health vulnerability is structurally produced rather than individually chosen (5), our findings identify *anticipatory governance of fear* as a key mechanism through which these structural conditions become operative in daily life. Migrants do not only encounter the state through police or immigration raids. Instead, the state appears in fragmented ways, shaping food practices through fear, anticipation, and self-regulation. In this sense, food emerges not merely as a site where structural violence is experienced, but as a domain where governance is lived, negotiated, and embodied.

Our findings reveal that in early 2024, a ‘pre-Trump 2.0 threshold’ environment had already psychologicalized the border. The ‘fear of speaking out’ and the withdrawal from public food spaces were not reactive responses to the 2025 raids, but rather a structural ‘anticipatory silence’. This reinforces the argument that deportability acts as a disciplinary tool that precedes actual police action, conditioning dietary and social agency months before the political escalation.

Building on Carney’s analysis of conditional incorporation and morally regulated food governance, this study makes a specific analytical intervention by foregrounding the temporal and spatial operation of governance under legal precarity. While Carney demonstrates how undocumented migrants are incorporated into state infrastructures in fragmented and conditional ways, our findings show that governance also operates *before* direct encounters with institutions or enforcement. Anticipatory governance of fear leads migrants to self-regulate preemptively, shaping daily food practices through shortened shopping routines, avoidance of public or institutional food spaces, and efforts to remain invisible. This temporal anticipation reveals how governance is enacted through micro-geographies of food access, where routes, vendors, and shopping rhythms function as techniques of everyday risk management under deportability.

It is important to note that the fieldwork for this study was conducted prior to the large-scale protests and public mobilizations that later emerged in states such as California, Chicago, and Minnesota in response to intensified immigration enforcement. Despite the absence of visible mass protest at the time, fear was already pervasive and deeply embedded in everyday life. Participants were often reluctant to be interviewed, limited their movements in public space, and expressed distrust even toward researchers who shared their national background. This ethnographic context underscores the anticipatory nature of governance documented in this study: fear did not emerge as a reaction to a specific event, but as an accumulated condition that structured daily practices well before overt conflict or collective mobilization became visible.

Read through this lens, practices of dietary invisibilization should not be understood as withdrawal from the state, but as a relational engagement with a state that is simultaneously punitive, necessary, and unevenly present. Migrants are not outside the state; they are incorporated in precarious, partial, and morally regulated ways, particularly through food assistance, health infrastructures, and informal economies (9, 10). In this context, invisibilization functions as a strategy for navigating a fragmented state presence, minimizing exposure to surveillance while remaining selectively connected to resources perceived as less risky or more familiar. Food practices thus become a key site where migrants manage conditional belonging and legal vulnerability in everyday life.

Beyond shaping spatial practices, anticipatory governance of fear becomes inscribed on the body itself, transforming health outcomes into an archive of political exclusion. Participants’ accounts of weight gain, chronic fatigue, hypertension, and prediabetes are not merely biomedical consequences of dietary change, but corporeal traces of governance operating through fear, anticipation, and sustained self-regulation. Building on Carney’s work, the body emerges here as the site where legal precarity, moral discipline, and nutritional constraint sediment over time (9, 22). From this perspective, chronic illness is not an unintended byproduct of migration, but an embodied manifestation of anticipatory governance produced through shortened provisioning circuits, dietary invisibilization, and prolonged stress. Governance thus extends beyond borders and institutions to act directly upon migrant bodies, where political exclusion is lived, metabolized, and reproduced in everyday life.

These findings also engage nutritional transition theory (12) through a justice-oriented lens. Obesity and increased consumption of ultra-processed foods among migrants emerge not from voluntary adoption, but from economic, temporal, and legal constraints that structure everyday food choices. At the same time, culture mediates this process. Migrant women in particular emerge as transnational caregivers, combining knowledge, memory, and resources across borders to sustain family health under conditions of scarcity (9). Through community networks and culinary traditions, they enact practices of care and resistance, echoing Bowen et al.’s (25) framing of food as a site of everyday resilience.

Narratives of preserving traditional foodways further reveal how migrants use culinary practices to sustain identity and belonging. One participant explained, “With the tamales I make […] I just do my best to make them still taste like Mexico,” illustrating efforts to preserve cultural flavors despite resource constraints. This resonates with Vázquez-Medina’s (2, 17) concept of transmigrant food identity, in which food becomes a site of symbolic resistance and cultural continuity. Traditional foodways thus appear not as static relics of the past, but as dynamic resources through which migrants reclaim dignity and meaning in contexts of marginalization.

These practices also align with Gálvez’s (14) notion of transnational food reciprocity. Participants’ accounts illustrate how the nutritional transition unfolds in lived experience. As one migrant noted, “When I got here, I started eating hamburgers and fast food. I didn’t eat that in Mexico because there weren’t many places like that.” This testimony reflects broader structural patterns described by Popkin et al. (12) and Gálvez (14), whereby globalized food systems and local food environments promote the consumption of inexpensive, calorie-dense products. Far from reflecting individual preference, these dietary shifts reveal how migrants’ food choices are shaped by spatially and economically configured food environments.

Taken together, this interdisciplinary analysis connects structural determinants with cultural meanings and embodied experience. Acculturative stress emerges through family conflicts, food-related shame, and health concerns, underscoring the need for culturally sensitive and rights-based health policies. As Mintz (33) reminds us, food embodies memory and power; preserving Mexican cuisine thus becomes a practice of dignity and belonging. Public health interventions should build on this potential by supporting traditional food knowledge, community gardens, and intercultural kitchens rather than imposing standardized, culturally alien dietary models.

While qualitative and context-specific, this study speaks to broader patterns observed among Global South migrants, including precarity, constrained agency, and everyday resilience. Future research would benefit from comparative and longitudinal designs examining intergenerational change and the role of emotions such as fear, anxiety, and nostalgia in shaping food practices over time.

In sum, understanding migrant food and health requires a critical lens that bridges structural forces and intimate family realities. By weaving social theory with ethnographic voices, this study contributes to a more humane and analytically grounded understanding of migration, recognizing migrant bodies not merely as sites of deprivation, but as carriers of knowledge, memory, and dignity under conditions of anticipatory governance.

## 6 Conclusion

At the intersection of nostalgia and exclusion lie the everyday experiences of undocumented Mexican migrants in Los Angeles. Participants in this study consistently described longing for the flavors, routines, and sense of security associated with home, while simultaneously navigating a social environment that restricts access to stable work, healthy food, and basic healthcare. Through a critical ethnographic approach, this study has shown how diet, health, labor, and culture become deeply entangled in migrants’ daily lives, producing outcomes marked by both vulnerability and resilience.

The findings demonstrate that poor dietary patterns and adverse health outcomes among undocumented migrants are not the result of individual choices or cultural preferences, but of structural conditions shaped by legal precarity, economic insecurity, and fear. Heightened deportability and enforcement regimes limit access to services and public spaces, fostering food insecurity, chronic stress, and long-term health deterioration. Importantly, these effects are not confined to moments of direct state intervention. Rather, they emerge through anticipatory governance of fear, whereby the possibility of enforcement regulates everyday behavior and becomes embodied in food practices and health trajectories.

At the same time, migrants’ narratives reveal that culture functions not only as memory but as a resource for care and resistance. Through the preservation of recipes, food rituals, and community ties, migrants sustain forms of dignity and belonging that partially buffer the effects of exclusion. These practices do not eliminate vulnerability, but they allow migrants to actively negotiate their conditions of life within constrained circumstances. In this sense, food practices become a site where exclusion is lived, but also where agency and continuity are reasserted.

By situating migrants’ dietary practices within an anticipatory enforcement context, this study contributes a temporal and embodied perspective to the literature on food insecurity and migration. It shows that governance operates not only through policies and institutions, but through everyday anticipations that shape how people eat, move, and care for their bodies over time. This perspective helps illuminate how fear, time, and embodiment intersect in the production of migrant health inequalities.

The implications are both analytical and political. Addressing food insecurity among migrant communities requires moving beyond emergency assistance toward broader agendas of social inclusion and health justice. Policies and interventions grounded in migrants’ cultural food practices, such as support for traditional diets, community gardens, mobile clinics, and culturally competent healthcare, are essential. Undocumented migrants are integral to the urban fabric of cities like Los Angeles; their health and well-being are collective concerns. Ensuring access to adequate nutrition and healthcare is therefore not an act of charity, but a matter of social justice and human dignity.

## Data Availability

The raw data supporting the conclusions of this article will be made available by the authors, without undue reservation.

## References

[1] Abarca ME. Voices in the kitchen: Views of food and the world from working-class Mexican and Mexican American women Texas A&M University Press (2006).

[2] Vázquez-Medina JA. Cocina, nostalgia y etnicidad en restaurantes mexicanos de Estados Unidos. Barcelona: Editorial UOC (2016).

[3] Joassart-Marcelli P. The $16 taco: Contested geographies of food, ethnicity, and gentrification. Seattle, WA: University of Washington Press (2021).

[4] Rosales R. Fruteros: Street vending, illegality, and ethnic community in Los Angeles. Seattle, WA: University of California Press (2020).

[5] Quesada J Hart LK Bourgois P. Structural vulnerability and health: Latino migrant laborers in the United States. Med Anthropol. (2011) 30:339–62. doi: 10.1080/01459740.2011.576725, 21777121 PMC3146033

[6] De Genova NP. Migrant “illegality” and deportability in everyday life. Annu Rev Anthropol. (2002) 31:419–47. doi: 10.1146/annurev.anthro.31.040402.085432

[7] Nussbaum MC. Women and human development: The capabilities approach. Cambridge: Cambridge University Press (2000).

[8] Sen A. Development as freedom. New York, NY: Alfred A. Knopf (1999).

[9] Carney MA. Eating and feeding at the margins of the state: barriers to healthcare for undocumented migrant women and the ‘clinical’ aspects of food assistance. Med Anthropol Q. (2015) 29:196–215. doi: 10.1111/maq.12151, 25715903

[10] Carney MA. “Back there we had nothing to eat”: Mexican and central American households in the U.S. and transnational food security. Int Migr. (2017) 55:64–77. doi: 10.1111/imig.12293

[11] Popkin BM. Nutritional patterns and transitions. Popul Dev Rev. (1993) 19:138–57. doi: 10.2307/2938388

[12] Popkin BM Adair LS Ng SW. Global nutrition transition and the pandemic of obesity in developing countries. Nutr Rev. (2012) 70:3–21. doi: 10.1111/j.1753-4887.2011.00456.x, 22221213 PMC3257829

[13] Popkin BM. The nutrition transition and obesity: not inevitable. Obes Rev. (2022) 23:e13366. doi: 10.1111/obr.13366, 34632692 PMC8639733

[14] Gálvez A. Eating NAFTA: Trade, food policies, and the destruction of Mexico. Oakland, CA: University of California Press (2018).

[15] Hilmers A Hilmers DC Dave J. Neighborhood disparities in access to healthy foods and their effects on environmental justice. Am J Public Health. (2012) 102:1644–54. doi: 10.2105/AJPH.2012.300865, 22813465 PMC3482049

[16] Fischler C. Food, self and identity. Soc Sci Inf. (1988) 27:275–93. doi: 10.1177/053901888027002005

[17] Vázquez-Medina JA. "No es comida para güeros: Resistencia alimentaria e identidad alimentaria transmigrante en establecimientos de comida mexicana en Estados Unidos" In: Medina FX, editor. Alimentación y migraciones en Latinoamérica. Barcelona, Spain: Editorial UOC (2014). 91–109.

[18] de Haas H. A theory of migration: the aspirations–capabilities framework. Comp Migr Stud. (2021) 9:8. doi: 10.1186/s40878-020-00210-4, 33680858 PMC7902564

[19] Denova-Gutiérrez E Flores YN Gallegos-Carrillo K Ramírez-Palacios P Rivera-Paredez B Barquera S . Impact of acculturation level and socioeconomic status on obesity and glucose metabolism in Mexican-American children. Am J Public Health. (2016) 106:70–3. doi: 10.2105/AJPH.2015.302923, 26562122 PMC4695951

[20] Pérez-Escamilla R Putnik P. The acculturation process and health status of Latinos in the USA: a review of the literature. Soc Sci Med. (2007) 64:982–96. doi: 10.1016/j.socscimed.2006.10.009

[21] Berry JW. Acculturation: living successfully in two cultures. Int J Intercult Relat. (2005) 29:697–712. doi: 10.1016/j.ijintrel.2005.07.013

[22] Carney MA Krause KC. Immigration/migration and healthy publics: the threat of food insecurity. Human Soc Sci Commun. (2020) 6:93. doi: 10.1057/s41599-020-0461-0

[23] Patel R. What does food sovereignty look like? J Peasant Stud. (2009) 36:663–706. doi: 10.1080/03066150903143079

[24] Joassart-Marcelli P Rossiter JS Bosco FJ. Ethnic markets and community food security in an urban “food desert”. Environ. Plan. (2017) 49:1642–63. doi: 10.1177/0308518X17700394

[25] Bowen S Elliott S Brenton J. The politics of food insecurity and migrant communities in the United States. J Migrat Health. (2023) 7:100050. doi: 10.1016/j.jmh.2023.100050

[26] Brones A. Food apartheid: The root of the problem with America’s groceries. The Guardian (2018). https://www.theguardian.com/society/2018/may/15/food-apartheid-food-deserts-racism-inequality-america-karen-washington-interview

[27] Levitt P. Social remittances: Migration driven local-level forms of cultural diffusion.Int Migr Rev. (1998) 32:926–48. doi: 10.1177/019791839803200404 12294302

[28] Moustakas C. Phenomenological Research Methods. Sage Publications (1994).

[29] Madison DS. Critical Ethnography: Method, Ethics, and Performance. 2nd edn. Sage Publications (2012).

[30] Patton MQ. Qualitative Research & Evaluation Methods: Integrating Theory and Practice. 4th edn. Sage Publications (2015).

[31] Charmaz K. Constructing Grounded Theory. 2nd edn. Sage Publications (2014).

[32] Braun V Clarke V. Thematic Analysis: A Practical Guide. Sage Publications (2021).

[33] Mintz SW. Sweetness and Power: The Place of Sugar in Modern History. Viking Penguin (1985).

